# Clinical utility of biomarkers of endothelial activation in sepsis-a systematic review

**DOI:** 10.1186/cc11145

**Published:** 2012-01-16

**Authors:** Katharine Xing, Srinivas Murthy, W Conrad Liles, Jeffrey M Singh

**Affiliations:** 1 Division of Hematology, University of British Columbia, Vancouver General Hospital, 855 12th Ave W, Vancouver, BC V5Z 1M9, Canada; 2 Divisions of Pediatric Infectious Disease and Critical Care, University of Toronto, Hospital for Sick Children, 555 University Avenue, Toronto, ON M5G 1X8, Canada; 3 McLaughlin-Rotman Centre for Global Health, University Health Network, 101 College Street, Suite 406 Toronto, ON M5G 1L7, Canada; 4 Department of Medicine, University of Toronto, 1 King's College Circle Medical Sciences Building-Room 2109, Toronto, ON M5S 1A8, Canada; 5 Interdepartmental Division of Critical Care Medicine, University of Toronto, Queen Street Wing, Room 4-042, 30 Bond Street, Toronto, ON M5B 1W8, Canada

**Keywords:** Sepsis, endothelium, biomarker, angiopoietin, coagulation

## Abstract

**Introduction:**

A strong biologic rationale exists for targeting markers of endothelial cell (EC) activation as clinically informative biomarkers to improve diagnosis, prognostic evaluation or risk-stratification of patients with sepsis.

**Methods:**

The objective was to review the literature on the use of markers of EC activation as prognostic biomarkers in sepsis. MEDLINE was searched for publications using the keyword 'sepsis' and any of the identified endothelial-derived biomarkers in any searchable field. All clinical studies evaluating markers reflecting activation of ECs were included. Studies evaluating other exogenous mediators of EC dysfunction and studies of patients with malaria and febrile neutropenia were excluded.

**Results:**

Sixty-one studies were identified that fulfilled the inclusion criteria. Overall, published studies report positive correlations between multiple EC-derived molecules and the diagnosis of sepsis, supporting the critical role of EC activation in sepsis. Multiple studies also reported positive associations for mortality and severity of illness, although these results were less consistent than for the presence of sepsis. Very few studies, however, reported thresholds or receiver operating characteristics that would establish these molecules as clinically-relevant biomarkers in sepsis.

**Conclusions:**

Multiple endothelial-derived molecules are positively correlated with the presence of sepsis in humans, and variably correlated to other clinically-important outcomes. The clinical utility of these biomarkers is limited by a lack of assay standardization, unknown receiver operating characteristics and lack of validation. Additional large-scale prospective clinical trials will be required to determine the clinical utility of biomarkers of endothelial activation in the management of patients with sepsis.

## Introduction

Sepsis is a complex syndrome that results from a host's response to invasive infection [1,2], and severe sepsis with organ dysfunction and septic shock are leading causes of death in critically ill patients [3]. A tool that would predict prognosis or allow risk-stratification of patients is needed to inform healthcare providers, families and decision makers, and facilitate the study and implementation of evolving therapeutic interventions.

A biomarker is defined as "...a characteristic that is objectively measured as an indicator of normal biological processes, pathogenic processes or pharmacologic responses to therapeutic intervention" [4]. Despite the proposal of over 100 distinct biological molecules as biomarkers for sepsis, no useful single biomarker, or combination thereof, has yet been identified [5].

**Figure 1:**
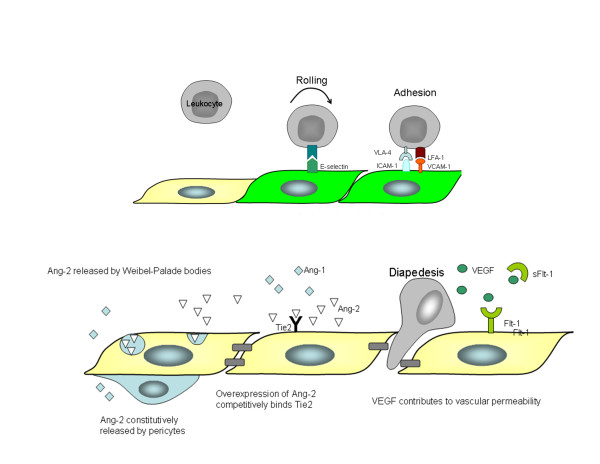
**Endothelial activation induces increased production of adhesion molecules such as ICAM-1, VCAM-1, E-selectin and P-selectin**. E-selectin induces leukocyte rolling, and ICAM-1 and VCAM-1 bind leukocyte function antigen 1 (LFA1) and very late antigen 4 (VLA4), respectively, to induce firm leukocyte adhesion. Activation is partially mediated by VEGF binding to VEGF receptor 1 (VEGFR1, also known as Flt-1) and VEGF receptor 2 (VEGFR2). Soluble Flt-1 binds VEGF competitively to render an anti-inflammatory response in the setting of sepsis. Ang-1 is constitutively secreted by pericytes and smooth muscle cells. Upon activation, Ang-2 is rapidly released by Weibel-Palade bodies, competitively interfering with Ang-1/Tie2 signaling and thereby increasing expression of adhesion molecules.

A hallmark of sepsis is a change in microvascular function. Widespread endothelial damage and apoptosis appears to be directly involved (see Figure 1), with numerous associations observed between sepsis and endothelial cell (EC) activation [6-10]. Consequently, there is a strong biologic rationale for targeting markers of endothelial activation as biomarkers of sepsis. A large number of EC-active molecules have been investigated as potential biomarkers for the early diagnosis, triage and prognostication of sepsis. These include regulators of endothelial activation, such as vascular endothelial growth factor (VEGF), endocan and the angiopoeitin pathway (Ang-1/2), adhesion molecules such as s-ICAM-1, sVCAM-1, and sE-selectin-1), mediators of permeability and vasomotor tone (s-Flt and endothelin-1); and mediators of coagulation (vWF, ADAMTS13).

Given the potential for, and growing interest in, EC-derived molecules as biomarkers in sepsis, we conducted a systematic review of the current published literature of biomarkers to determine their performance in predicting the severity of sepsis and clinical outcomes. This systematic review will serve as an update and supplement to other recent reviews in the literature [5,11-14], given the rapidly evolving nature of the field.

## Materials and methods

### Data sources

We systematically and inclusively identified all studies evaluating markers of endothelial activation, (including angiopoietins and sTie2R, sVEGF and sFlt-1, sICAM-1, sVCAM-1, sE-selectin, endothelin-1, endocan, VWF and ADAMTS13) in sepsis. We electronically searched MEDLINE (1950 to Week 2, September 2011) and EMBASE (1980 to Week 37, 2011) databases for all pertinent English language studies. (Please see Additional file 1, Search Strategy).

### Study selection methods

Study selection was performed independently by three reviewers (KX, SM, JMS), with disagreements resolved through arbitration by a fourth reviewer (WCL). A study was included if it (1) studied adult patients with sepsis or the systemic inflammatory response syndrome (SIRS), or studied patients at risk for sepsis or SIRS, and (2) evaluated a clinical endpoint (the development of sepsis, sepsis severity, development of organ dysfunction or mortality). Studies of patients less than 18 years of age, patients with febrile neutropenia, patients with malaria, interventional clinical trials studying a specific intervention or medication and case reports were excluded.

### Study data extraction and analysis

For each of the selected studies, we extracted the biomarker(s) evaluated, study size and patient population, and details of the primary and secondary outcomes. Outcomes of interest for each biomarker were tabulated and compared across studies where appropriate. Study design, standardization of sepsis definition and other methodological data were extracted and each study was subject to the Grading of Recommendations Assessment, Development and Evaluation (GRADE) system for assessing the quality of evidence [15]. Due to the anticipated broad study heterogeneity and disparate study outcomes, we did not attempt to numerically combine or perform a meta-analysis of study results.

## Results

**Figure 2:**
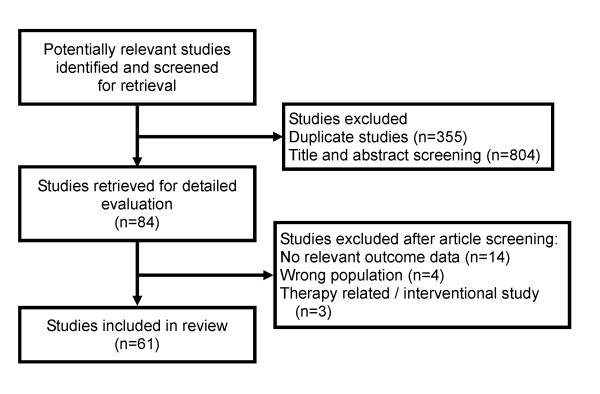
**Study flow diagram**.

Our search identified 1,243 unique articles (see Figure 2). A total of 84 studies met our predefined inclusion and exclusion criteria, of which a further 23 studies were excluded after retrieval of full-text publication for the following reasons: 14 studies did not report a clinical outcome [16-29], 4 studies did not include a relevant patient population [30-32], 3 studies were interventional trials [33-35], and 2 studies were not in English and the English abstracts provided insufficient information to allow adjudication of study inclusion [36,37]. The remaining 61 studies were included in our review.

All studies were observational designs, including secondary analyses of data collected during prospective clinical trials. Most studies used standard consensus definitions of sepsis. Interpretation of the magnitude of effect or association between biomarkers and sepsis or clinical outcomes was limited by a lack of standardization in individual biomarker assays, an absence of identified or validated thresholds or cut-points in individual biomarker levels, and a lack of reported odds ratios or relative risk. Several studies identified positive associations between biomarker levels and severity of sepsis (for example, sepsis, severe sepsis and septic shock), but given the aforementioned limitations and heterogeneity across studies in this association, we did not deem this to be sufficient evidence of a dose-response association to upgrade the quality level of these studies given the aforementioned limitations. Consequently, all studies were assigned a GRADE level of 'low quality' with respect to the association between individual biomarker levels and sepsis [15].

### The Angiopoietin system

We identified 11 studies investigating angiopoietin 2 (Ang-2) as a biomarker in human sepsis (see Table 1- Studies Evaluating Angiopoietin-2). All but one were prospective observational studies [38-44], with one secondary analysis of a previously conducted cohort study [45].

**Table 1:** Studies evaluating angiopoietin-2

Study	Year	N	Population	Standard Criteria for SIRS/Sepsis	Association with Sepsis	Other Outcomes
Parikh *et al*., [43]	2006	51	ICU patients with sepsis (22) and hospitalized controls (29)	2003 ACCP/SCCM [2]	Ang-2 higher in patients severe sepsis than patients with sepsis and controls (23.2 vs. 4.8 and 3.5 ng/mL respectively; *P *< 0.01)	
Van der Heijden *et al*., [45]	2008	112	Mechanically ventilated patients, with sepsis (24) and without (88)	1992 ACCP/SCCM [1]	Ang-2 higher in patients with sepsis than without sepsis (4.1 vs. 0.4 ng/mL; *P *< 0.01)	Higher Ang-2 associated with ALI/ARDS (*P *< 0.001) and higher in ARDS than in ALI (*P *> 0.001); Independently associated with the severity of pulmonary leak (r = 0.41; *P *= 0.014).
Orfanos *et al*., [38]	2007	61	ICU patients	1992 ACCP/SCCM [1]	Ang-2 higher in severe sepsis compared to patients without SIRS or sepsis (*P *< 0.05 by analysis of variance)	Ang-2 levels correlated with levels of circulating TNF (*P *< 0.05)
Giamarellos-Bourboulis *et al*., [40]	2008	60	Trauma patients admitted to ICU (54) and healthy controls (6)	2003 ACCP/SCCM [2]	Ang-2 higher in sepsis and severe sepsis than in healthy controls, or trauma patients with sterile SIRS (*P *< 0.05); Predictive of sepsis/severe sepsis (*P *= 0.017, 0.002 respectively); Increases in serial Ang-2 predicted development of sepsis (*P *< 0.05)	Ang-2 correlated with 28-day survival (*P *= 0.015)
Kumpers *et al*., [42]	2008	72	Patients admitted to medical ICU (43) and healthy controls (29)	2003 ACCP/SCCM [2]	Ang-2 higher in septic patients than in patients without sepsis (16.5 vs. 2.8 ng/mL; *P *< 0.001); Not correlated with severity of sepsis (median Ang-2 16.5 vs. 28.1 ng/mL; *P *= 0.12);	Ang-2 correlated with mortality (*P *= 0.001)
Davis *et al*., [44]	2010	124	Patients admitted to a mixed ICU	1992 ACCP/SCCM [1]	Ang-2 higher in patients with severe sepsis compared to patients with sepsis without organ failure and non-septic controls (12.4 vs. 6.1 and 2.7 ng/mL, respectively; *P *< 0.0001).	Ang-2 not predictive of 28-day mortality (*P *= 0.32)
Siner *et al*., [39]	2009	66	Patients admitted to ICU	1992 ACCP/SCCM [1]	Ang-2 not correlated with severity of sepsis	Ang-2 correlated with mortality (*P *= 0.02)
Ricciuto *et al*. [48]	2011	70	Patients with severe sepsis	1992 ACCP/SCCM [1]		Admission levels of Ang-2 and Ang-2/Ang-1 ratio were not associated with 28-day mortality Serially measured Ang-2 levels correlated directly with the MOD score (*P *= .003)
Ebihara *et al*. [49]	2011	25	25 patients treated with Polymyxin-B column hemoperfusion 11 developed ALI	1992 ACCP/SCCM [1]		Positive correlation between Ang-1 and PaO2/FiO2 ratio (r = 0.427; *P *< 0.001) Inverse correlation between Ang-2 and PaO2/FiO2 ratio (r = 0.302; *P *= 0.003)
Page *et al*., [50]	2011	37	16 invasive streptococcal infection and toxic shock 21 invasive steptococcal infection alone	*S. pyogenes *isolated from normally sterile site and 2009 Consensus definition of streptococcal toxic shock	Ang-2:Ang-1 ratio increased in Streptococcal Toxic Shock Syndrome compared to those with uncomplicated invasive streptococcal infection (*P *< 0.05)	
Kranidoti *et al*., [41]	2009	107	ICU patients with Ventilator Associated pneumonia (90) and healthy controls (17)	2003 ACCP/SCCM [1]	Ang-2 higher in septic patients compared to healthy controls. (*P *< 0.001)	Ang-2 correlated with mortality (*P *< 0.05); Ang-2 levels greater than 9.7 ng/mL associated with sepsis-related mortality (OR = 3.3; *P *= 0.033)

ACCP, American College of Chest Physicians; ALI, Acute Lung Injury; APACHE II, Acute Physiology and Chronic Health Evaluation II; ARDS, Acute Respiratory Distress Syndrome; ED, emergency department; MOF, Multiple Organ Failure; SAPS, Simplified Acute Physiology Score; SCCM, Society of Critical Care Medicine; SIRS, systemic inflammatory response syndrome; SOFA, Sequential Organ Failure Assessment

#### Association with sepsis

Seven studies evaluated the association between Ang-2 levels and sepsis, reporting higher levels of Ang-2 in patients with sepsis compared to patients without sepsis in the ward setting [43,44], the ICU [38,40,42,45], and patients with acute lung injury/acute respiratory distress syndrome (ALI/ARDS) [45]. Ang-2 levels were also higher in sepsis than in either patients with sterile SIRS [40] or healthy controls [41]. Kumpers *et al*. also reported that Ang-2 concentrations were elevated in all ICU patients (irrespective of sepsis status) compared to healthy controls [42]. One study found that patients who did not have SIRS/sepsis on admission but subsequently developed SIRS/sepsis, had significant increases in Ang-2 over time [40].

There were inconsistent reports of the association between Ang-2 and the severity of sepsis (as defined by sepsis, severe sepsis and septic shock), with one positive study [44] and four studies that failed to observe a consistent correlation [38,39,41,42]. Higher levels of Ang-2 were also reported in patients with severe sepsis compared to septic ICU patients without organ dysfunction [38,43,44], non-septic hospitalized controls [43,44], and ICU patients without SIRS [38].

None of the studies identified a cut point or threshold of circulating Ang-2 that allowed differentiation of patients with sepsis and without sepsis, or stratification of patients with respect to sepsis severity based on baseline or serial serum Ang-2 concentrations.

#### Association with clinical outcome

Three studies [39,40,42] observed associations between circulating Ang-2 levels and severity of illness as defined by Acute Physiology and Chronic Health Evaluation II (APACHE II) [46] or Sequential Organ Failure Assessment score (SOFA) [47], and five studies reported a relationship between increasing Ang-2 levels and increasing mortality [39-42,48]. Kumpers *et al*. found that circulating Ang-2 levels were independently associated with 30-day survival after adjustment for APACHE II score, SOFA score and serum lactate levels [42]. Kranidioti *et al*. found that Ang-2 concentrations were associated with sepsis-related mortality at baseline and every day for the first seven days in ICU, and Ang-2 levels greater than 9.7 ng/mL were associated with a three-fold increased risk of sepsis-related mortality [41]. Siner *et al*. found higher Ang-2 levels were associated with hospital motality, and the patient cohort could be stratified for hospital mortality by admission Ang-2 levels [39]. Ricciuto *et al*. observed that serial measurements of Ang-2 were associated with 28-day mortality and multiple organ dysfunction (MOD) score [48].

One study found Ang-2 was independently associated with the severity of lung injury as measured by pulmonary leak, and was predictive for the development of ARDS [45]. A second study found an inverse correlation between Ang-2 and PaO2/FiO2 ratio [49]. Page *et al*. found that the Ang-2/Ang-1 ratio was significantly increased in patients with invasive streptococcal infection who developed toxic shock syndrome, compared to those with uncomplicated infection [50].

### The leukocyte adhesion pathway

We identified 19 studies investigating sICAM-1 as a sepsis biomarker (see Table 2-Studies evaluating sICAM), 12 studies for sVCAM-1 (see Table 3-Studies Evaluating sVCAM-1), 23 studies for sE-selectin-1 (Table 4-Studies Evaluating sE-selectin-1), and 2 studies for endocan (see Table 5-Studies Evaluating Endocan). All were prospective studies or secondary analyses of prospective studies. These studies focused on emergency room patients with suspected infections or shock [51,52], and critically ill patients admitted to intensive care units, including medical and surgical patients [51,53-76], patients with ventilator-associated pneumonia (VAP) [73], trauma [62,63,66,67,75], and post-cardiopulmonary resuscitation [74].

**Table 2:** Studies evaluating sICAM

Study	Year	N	Population	Standard Criteria for SIRS/Sepsis	Associations with sepsis	Other outcomes
Shapiro *et al*., [51]	2010	221	ED patients with sepsis without organ dysfunction (71), severe sepsis without shock (66), septic shock (71), and non-infected controls (13)	1992 ACCP/SCCM [1]	sICAM-1 elevated in septic shock compared with non-infected controls (*P *< 0.05);	sICAM-1 associated with increasing sepsis severity *P *< 0.05; modest correlation with SOFA and APACHE-II; predicts mortality and severe sepsis (AUC of 0.72 (95% CI 0.57 to 0.87), 0.61 (95% CI 0.53 to 0.69))
Schuetz *et al*., [52]	2011	161	Patients with hypotension: 69 sepsis 35 cardiac 12 hemorrhagic 12 unknown	Clinical classification based on clinical and microbiological data	ICAM-1 higher in sepsis compared to non-sepsis (*P *< 0.05)	
Hofer *et al*., [55]	2009	147	Surgical ICU patients with severe sepsis (101) and major abdominal surgery (28), and healthy controls (18)	2003 ACCP/SCCM [2]	sICAM-1 higher in the septic group than postoperative and volunteer groups at diagnosis (444.7 ng/ml vs 213.7 ng/ml and 219.6 ng/ml, respectively; *P *< 0.001)	Not predictive of mortality at the time of diagnosis of sepsis, but non-survivors had trend to higher sICAM-1 levels at 48 h and 120 h (683.2 vs 434.1 ng/ml, *P *= 0.067; 360.2 vs 467.8 ng/ml, *P *= 0.083, respectively) compared to survivors
Stief *et al*., [54]	2007	86	ICU patients with Sepsis (62), healthy controls (24)	Clinical definition of sepsis	Higher in sepsis than controls (2.56 ug/ml vs 0.19 ug/ml; *P *< 0.05)	
Scherpereel *et al*., [53]	2006	90	ICU patients with sepsis (63), SIRS (7), healthy controls (20)	1992 ACCP/SCCM [1]	sICAM-1 higher in sepsis compared to SIRS *P *< 0.02	sICAM-1 not predictive of mortality or severity of sepsis
Kinoshita *et al*., [56]	2002	56	Gram negative sepsis from intra-abdominal infection admitted to surgical ICU (47), healthy controls (9)	1992 ACCP/SCCM [1]	sICAM-1 higher in sepsis than healthy controls	Not correlated with mortality in those with ARDS; Higher in those with ARDS than those without *P *< 0.05
Paterson *et al*., [57]	2000	16	ICU patients with SIRS (10), healthy controls (6)	1992 ACCP/SCCM [1]	sICAM-1 not reported in healthy controls	Not correlated with mortality
Weigand *et al*., [58]	1999	21	Surgical ICU patients with septic shock (14), healthy controls (7)	1992 ACCP/SCCM [1]	sICAM-1 significantly higher in sepsis than controls (*P *< 0.05)	sICAM-1 significantly higher in nonsurvivors than survivors, sensitivity and specificity for cutoff of 800 ng/ml was 74.1%
Froon *et al*., [73]	1998	42	ICU patients with sepsis and VAP	1992 ACCP/SCCM [1]	sICAM-1 higher in VAP patients complicated by severe sepsis or septic shock than other VAP patients, but statistical significance not achieved	Not predictive of mortality, and correlates poorly with SAPS-II (r = 0.16, *P *= 0.30)
Kayal *et al*., [59]	1998	41	ICU patients with severe sepsis or septic shock (25), ICU controls (7), healthy controls (9)	1992 ACCP/SCCM [1]	sICAM-1 higher in septic patients than in noninfected ICU controls and healthy volunteers (*P *< 0.0001); higher in septic shock than those without septic shock (*P *< 0.05)	sICAM-1 correlated with mortality; correlated with SAPS and MOF score (r = 0.53, *P *< 0.01 for MOF)
Boldt *et al*., [60]	1997	30	Surgical ICU patients with post-operative sepsis (30), healthy controls (not stated)	1992 ACCP/SCCM^1^	sICAM-1 higher in septic patients than healthy controls	Higher in older than younger patients *P *< 0.05, and tends to increase in older patients and decrease in younger patients over time
Egerer *et al*., [61]	1997	24	ICU patients with infection (8), severe sepsis (16)	1992 ACCP/SCCM [1]	sICAM-1 higher in severe sepsis compared with patients with infection (*P *> 0.05)	Not correlated with mortality in patients with severe sepsis
Takakuwa *et al*., [62]	1997	34	ICU admissions with sepsis (20), trauma (14)	Clinical definition of SIRS and sepsis	sICAM-1 level higher in septic than trauma patients (987.7 vs 472.1 ng/ml; *P *= 0.0002)	sICAM-1 correlated with endotoxin, TNF-α, IL-6, IL-8, Type II PLA2 (Type II phospholiaps A2), NO (*P *< 0.05 for all)
Boldt *et al*., [63]	1996	30	Surgical ICU patients with postoperative sepsis (15), trauma (15)	1992 ACCP/SCCM [1]	sICAM-1 higher in sepsis than trauma (1,266 vs 444 ng/ml; *P *< 0.01)	
Endo *et al*., [64]	1996	28	ICU patients with sepsis with MOF (8), sepsis without MOF (15), MOF without sepsis (5)	Clinical diagnosis of sepsis	sICAM-1 higher in septic patients with or without MOF than patients with MOF but no infection (1103.3 vs 356.0 ng/ml, and 862.5 vs 356.0 ng/ml, respectively, *P *< 0.05))	sICAM-1 level higher in septic patients with MOF than those without (*P *= 0.0401)
Moss *et al*., [66]	1996	55	ICU patients with sepsis (19), trauma (36) controls (5)	Clinical diagnosis of sepsis	sICAM-1 higher in septic patients than trauma and controls (573 vs 148 and 235 ng/ml, respectively, *P *< 0.001)	
Nakae *et al*., [67]	1996	34	ICU patients with sepsis (21), trauma (13)	1992 ACCP/SCCM [1]	sICAM-1 higher in septic patients than in trauma patients (987 vs 472 pg/ml; *P *= 0.0002)	sICAM-1 correlated with endotoxin, TNF-alpha and IL-8 (*P *< 0.05 for all)
Sessler *et al*., [68]	1995	66	ICU patients with sepsis (25), SIRS (25), ICU controls (4), healthy volunteers (12)	1992 ACCP/SCCM [1]	sICAM-1 higher in sepsis than ICU controls and healthy controls (1,259 vs 585 ng/ml, *P *< 0.001; 1,259 vs 355 ng/ml, *P *< 0.0001); sICAM-1 is higher in SIRS than ICU controls and healthy controls (937 vs 585 ng/ml, *P *< 0.05; 937 vs 355 ng/ml, *P *< 0.001); higher in sepsis vs SIRS (1,259 vs 937 ng/ml; *P *= 0.12)	sICAM-1 elevated with increasing severity of illness: septic shock, severe sepsis and sepsis (1,551, 796, and 542 ng/ml, respectively, ANOVA *P *= 0.017); correlated with cumulative MOF score, shock severity score (r = 0.46, *P *= 0.021; r = 0.50, *P *< 0.009); higher in nonsurvivors vs survivors (1,697 vs 854 ng/ml; *P *= 0.0096)
Cowley *et al*., [65]	1994	125	ICU patients with sepsis (21), severe sepsis (14), ICU controls (5), healthy controls (85)	Clinical definition of SIRS and sepsis	sICAM-1 higher in severe sepsis, uncomplicated sepsis, and ICU controls than healthy controls *P *< 0.05.	sICAM-1 with no significant difference between severe sepsis, uncomplicated sepsis and ICU controls. Not correlated with mortality

ACCP, American College of Chest Physicians; ALI, Acute Lung Injury; APACHE II, Acute Physiology and Chronic Health Evaluation II; ARDS, Acute Respiratory Distress Syndrome; ED, emergency department; MOF, Multiple Organ Failure; SAPS, Simplified Acute Physiology Score; SCCM, Society of Critical Care Medicine; SIRS, Systemic Inflammatory Response Syndrome; SOFA, Sequential Organ Failure Assessment

**Table 3:** Studies evaluating sVCAM-1

Study	Year	N	Population	Standard Criteria for SIRS/Sepsis	Association with sepsis	Other outcomes
Shapiro *et al*., [51]	2010	221	ED patients with sepsis without organ dysfunction (71), severe sepsis without shock (66), septic shock (71), and non-infected controls (13)	1992 ACCP/SCCM [1]	sVCAM-1 elevated in septic shock compared with non-infected controls (*P *< 0.05)	sVCAM-1 associated with sepsis severity *P *< 0.04; predicts mortality and severe sepsis (AUC of 0.57 (95% CI 0.35 to 0.79), 0.60 (95% CI 0.52 to 0.69))
Hofer *et al*., [55]	2009	147	Surgical ICU patients with severe sepsis (101), major abdominal surgery (28), healthy controls (18)	2003 ACCP/SCCM [2]	sVCAM-1 did not differentiate between septic, postoperative and healthy controls	sVCAM-1 not predictive of mortality at the time of diagnosis of sepsis, but nonsurvivors had elevated sVCAM-1 at 48 h and 120 h compared to survivors(1,275.1 vs 882.0 ng/ml, *P *= 0.027; 1,685.5 vs 748.5 ng/ml; *P *= 0.021, respectively)
Kinoshita *et al*., [56]	2002	56	Gram negative sepsis from intra-abdominal infection admitted to surgical ICU (47), healthy controls (9)	1992 ACCP/SCCM [1]	sVCAM-1 higher in patients than healthy controls	sVCAM-1 did not differentiate those with ARDS from those without; not predictive of mortality in those with ARDS
Presterl *et al*., [69]	1999	40	ICU patients with Candida (20) and bacterial sepsis (20)	1992 ACCP/SCCM [1]	At all times (days 1, 7, 14) sVCAM-1 levels higher in Candida sepsis than bacterial sepsis (*P *< 0.05)	sVCAM-1 not correlated with mortality
Knapp *et al*., [78]	1998	54	Patients with sepsis (28 gram positive, 11 gram negative), 15 healthy controls	1992 ACCP/SCCM [1]	sVCAM-1 elevated in sepsis compared with healthy controls (*P *< 0.05)	sVCAM-1 does not correlate with mortality in gram positive sepsis; does not distinguish between gram positive and gram negative sepsis
Boldt *et al*., [60]	1997	30	Surgical ICU patients with post-operative sepsis (30), healthy controls (not stated)	1992 ACCP/SCCM [1]	sVCAM-1 higher in septic patients than healthy controls	Higher in older than younger patients *P *< 0.05, and tends to increase in older patients and decrease in younger patients over time
Takakuwa *et al*., [62]	1997	34	ICU admissions with sepsis (20), trauma (14)	Clinical definition of SIRS and sepsis	sVCAM-1 higher in septic than trauma patients (2,536 vs 1,019.0 ng/ml; *P *= 0.0004)	sVCAM-1 level correlated with TNF-α, IL-6, IL-8, NO, sE-selectin-1 ((*P *< 0.05 for all)
Boldt *et al*., [63]	1996	30	Surgical ICU patients with postoperative sepsis (15), trauma (15)	1992 ACCP/SCCM [1]	sVCAM-1 is higher in sepsis than trauma (1,042 vs 689 ng/ml; *P *< 0.05)	
Endo *et al*., [64]	1996	28	ICU patients with sepsis with MOF (8), sepsis without MOF (15), MOF without sepsis (5)	Clinical diagnosis of sepsis	sVCAM-1 higher in septic patients with or without MOF than patients with MOF but no infection (2,654.9 vs 945.0 ng/ml, *P *= 0.0295; 2,045.0 vs 945.0 ng/ml, *P *= 0.0037)	sVCAM-1 did not differ between septic patients with and without MOF (2,654.9 vs 2,045.0 ng/ml; *P *= 0.1315)
Furian *et al*., [76]	2011	45	Patients admitted to ICU with severe sepsis or septic shock	1992 ACCP/SCCM [1]		sVCAM-1 not associated with left ventricular function or size.
Schuetz *et al*., [52]	2011	161	Patients with hypotension: 69 sepsis, 35 cardiac, 12 hemorrhagic, 12 unknown	Clinical classification based on clinical and microbiological data	VCAM-1 higher in sepsis compared to non-sepsis (*P *< 0.05)	
Cowley *et al*., [65]	1994	125	ICU patients with sepsis (21), severe sepsis (14), ICU controls (5), healthy controls (85)	Clinical definition of SIRS and sepsis	sVCAM-1 is higher in sepsis than controls	sVCAM-1 higher in severe sepsis than uncomplicated sepsis at baseline (*P *= 0.06) and peak concentrations *P *< 0.01. Not correlated with mortality

ACCP, American College of Chest Physicians; ALI, Acute Lung Injury; APACHE II, Acute Physiology and Chronic Health Evaluation II; ARDS, Acute Respiratory Distress Syndrome; ED, emergency department; MOF, Multiple Organ Failure; SAPS, Simplified Acute Physiology Score; SCCM, Society of Critical Care Medicine; SIRS, Systemic Inflammatory Response Syndrome; SOFA, Sequential Organ Failure Assessment

**Table 4:** Studies evaluating sE-selectin-1

Study	Year	N	Population	Standard Criteria for SIRS/Sepsis	Association with sepsis	Other outcomes
Schuetz *et al*., [52]	2011	161	Patients with hypotension: 69 sepsis, 35 cardiac, 12 hemorrhagic, 12 unknown	Clinical classification based on clinical and microbiological data	E-selectin higher in sepsis compared to non-sepsis (*P *< 0.05) E-selectin independently associated with sepsis after adjustment for age, sex, blood pressure and mortality (*P *= 0.001) with AUC of 0.74 for discrimination of sepsis and non-sepsis	
Shapiro *et al*., [51]	2010	221	ED patients with sepsis without organ dysfunction (71), severe sepsis without shock (66), septic shock (71), and non-infected controls (13)	1992 ACCP/SCCM [1]	sE-selectin-1 levels elevated in septic shock compared with non-infected controls	sE-selectin-1 associated with sepsis severity *P *< 0.001; predicts mortality and severe sepsis (AUC of 0.65 (95% CI 0.49 to 0.82) and 0.71 (95% CI 0.64 to 0.78) respectively)
Stief *et al*., [54]	2007	86	ICU patients with Sepsis (62), healthy controls (24)	Clinically diagnosed sepsis	sE-selectin-1 elevated in sepsis compared to reference value (190 ng/ml vs 55 ng/ml; *P *< 0.05))	
Kinoshita *et al*., [56]	2002	56	Gram negative sepsis from intra-abdominal infection admitted to surgical ICU (47), healthy controls (9)	1992 ACCP/SCCM [1]	sE-selectin-1 does not differentiate between ARDS from non ARDS	Not predictive of mortality in those with ARDS
Geppert *et al*., [74]	2000	32	ICU patients on day two post successfulCPR (25), non-critically ill controls (7)	1992 ACCP/SCCM [1]	sE-selectin-1 higher in SIRS compared to controls (96.2 ng/ml vs 42.8 ng/ml; *P *= 0.23), but does not differentiate patients with SIRS vs patients without SIRS	Higher in non-survivors than survivors (114.2 ng/ml vs 85.7 ng/ml; *P *= 0.025)
Osmanovic *et al*., [72]	2000	27	ICU patients with sepsis with MOF (9), healthy controls (18)	Clinical definition of sepsis	sE-selectin-1 higher in sepsis compared to healthy controls (118 vs 28.5 ng/ml; *P *< 0.001)	
Hynninen *et al*., [70]	1999	20	ICU patients with severe sepsis (11), severe acute pancreatitis (9)	1992 ACCP/SCCM [1]	sE-selectin does not differentiation between those with severe acute pancreatitis and severe sepsis	Higher in those with higher SOFA scores (SOFA ≥ 10, *P *= 0.043), but not correlated with mortality
Presterl *et al*., [69]	1999	40	ICU patients with candida (20) and bacterial sepsis (20)	1992 ACCP/SCCM [1]	sE-selectin-1 lower in patients with Candida sepsis than bacterial sepsis (*P *< 0.05) on Day 1	Higher in non-survivors
Takala *et al*., [71]	1999	76	Hospitalized patients with sepsis with organ failure (8) and without organ failure (12); healthy controls (56)	1992 ACCP/SCCM [1]	sE-selectin-1 level elevated in septic patients compared to healthy adults *P *< 0.001	Not correlated with organ failure
Weigand *et al*., [58]	1999	21	Surgical ICU patients with septic shock (14), healthy controls (7)	1992 ACCP/SCCM [1]	sE-selectin-1 higher in sepsis than healthy controls (*P *< 0.05)	Not predictive of mortality or severity of disease
Froon *et al*., [73]	1998	42	ICU patients with sepsis and VAP	1992 ACCP/SCCM [1]	sE-selectin-1 higher in patients with severe sepsis or septic shock than other VAP patients, but statistical significance not achieved	Day 2 sE-selectin-1 higher in nonsurvivors than survivors (114.3 vs 67.0 ng/ml; *P *= 0.04); Correlates poorly with SAPSII (r = 0.18, *P *= 0.25)
Kayal *et al*., [59]	1998	41	ICU patients with severe sepsis or septic shock (25), ICU controls (7), healthy controls (9)	1992 ACCP/SCCM [1]	sE-selectin-1 higher in septic patients than noninfected ICU controls and healthy volunteers (p < 0.0001); higher in those with septic shock than those without (p < 0.05)	sE-selectin-1 higher in nonsurvivors than survivors on day 0 (286 vs 195 ng/ml; *P *< 0.05), but decreases after Day 3 of sepsis to reach a level similar to that of survivors Day 14; correlated with SAPS and MOF score (r = 0.45, *P *< 0.05 for MOF)
Knapp *et al*., [78]	1998	54	Patients with sepsis (28 gram positive, 11 gram negative), 15 healthy controls	1992 ACCP/SCCM [1]	sE-selectin-1 higher in septic patients than controls p < 0.05	sE-selectin-1 higher in nonsurvivors than survivors of gram positive sepsis on day 0, 4 and 7 (175 vs 85 ng/ml, *P *< 0.01; 155.7 vs 78.8 ng/ml, *P *< 0.05; 180 vs 76.1 ng/ml, *P *< 0.001, respectively); does not differentiate gram positive from gram negative infections.
Boldt *et al*., [60]	1997	30	Surgical ICU patients with post-operative sepsis (30), healthy controls (not stated)	1992 ACCP/SCCM [1]	sE-selectin-1 higher in septic patients than healthy controls	Higher in older than younger patients *P *< 0.05, and tends to increase in older patients and decrease in younger patients over time
Cummings *et al*., [79]	1997	119	ICU patients with sepsis (67), SIRS (44), ICU controls (8)	1992 ACCP/SCCM [1]	sE-selectin-1 higher in culture positive sepsis than culture negative sepsis, SIRS and ICU controls (15.39 vs 4.87, 2.33, and 1.97 ng/ml, respectively; *P *< 0.0001)	Day 1 levels higher for nonsurvivors than survivors (10.61 vs 4.35 ng/ml of log transformed mean sE-selectin-1; *P *< 0.05); sE-selectin-1 correlates strongly to the degree of hemodynamic compromise (*P *< 0.0001), and moderately with the peak MOF score (r = 0.30, *P *= 0.001)
Egerer *et al*., [61]	1997	24	ICU patients with infection (8), severe sepsis (16)	1992 ACCP/SCCM [1]	Higher in patients with severe sepsis and MOF than those with infection alone (*P *< 0.05)	Higher in nonsurvivors than survivors on Day 7-8, *P *< 0.05
Takakuwa *et al*., [62]	1997	34	ICU admissions with sepsis (20), trauma (14)	No Standard Definition	sE-selectin-1 higher in sepsis than trauma (287.9 vs 195.0 ng/ml; *P *= 0.0055)	sE-selectin-1 level correlated with TNF-α, IL-8, Type II PLA2, sICAM-1 (*P *< 0.005 for all)
Boldt *et al*., [63]	1996	30	Surgical ICU patients with postoperative sepsis (15), trauma (15)	1992 ACCP/SCCM [1]	sE-selectin-1 higher in sepsis than trauma (340 vs 57.9 ng/ml; *P *< 0.05)	
Endo *et al*., [64]	1996	28	ICU patients with sepsis with MOF (8), sepsis without MOF (15), MOF without sepsis (5)	Clinical diagnosis of sepsis	sE-selectin-1 higher in septic patients with or without MOF than patients with MOF but no infection (345.2 vs 121.8 ng/ml, *P *= 0.0016; 266.2 vs 121.8 ng/ml, *P *= 0.0054)	sE-selectin-1 did not differ significantly between septic patients with and without MOF (345.2 vs 266.2 ng/ml; *P *= 0.2939)
Moss *et al*., [66]	1996	55	ICU patients with sepsis (19), trauma (36) controls (5)	Clinical diagnosis of sepsis	Higher in sepsis than trauma and controls (573 vs 148 and 235 ng/ml, respectively, *P *< 0.001)	
Simons *et al*., [75]	1996	50	Multiple trauma patients, infectious complications in 14	Not specified	sE-selectin-1 higher in patients who subsequently developed infection, organ dysfunction, or both, by 36 h. *P *= 0.08	sE-selectin-1 higher in non-survivors than survivors (*P *= 0.0018)
Cowley *et al*., [65]	1994	125	ICU patients with sepsis (21), severe sepsis (14), ICU controls (5), healthy controls (85)	Clinical definition of SIRS and sepsis	sE-selectin higher in sepsis than controls (*P *< 0.01).	sE-selectin-1 higher in severe sepsis than uncomplicated sepsis on presentation (*P *< 0.01) and more pronounced with peak values (*P *< 0.001). Not correlated with mortality
Newman *et al*., [80]	1993	88	ICU patients with sepsis with positive blood cultures (17), healthy controls (71)	Clinical definition of sepsis	Higher in septic shock than controls (23.3 vs 0.92 ng/ml; *P *< 0.05); not elevated in uncomplicated sepsis compared to controls	

ACCP, American College of Chest Physicians; ALI, Acute Lung Injury; APACHE II, Acute Physiology and Chronic Health Evaluation II; ARDS, Acute Respiratory Distress Syndrome; ED, emergency department; MOF, Multiple Organ Failure; SAPS, Simplified Acute Physiology Score; SCCM, Society of Critical Care Medicine; SIRS, Systemic Inflammatory Response Syndrome; SOFA, Sequential Organ Failure Assessment

**Table 5:** Studies evaluating Endocan

Study	Year	N	Population	Standard Criteria for SIRS/Sepsis	Association with sepsis	Other outcomes
Scherpereel *et al*., [53]	2006	90	ICU patients with sepsis (63), SIRS (7), healthy controls (20)	1992 ACCP/SCCM [1]	Higher in sepsis than SIRS or healthy controls (2.71 vs 0.77 and 0.68 ng/ml; *P *< 0.001);higher in septic shock than severe sepsis and uncomplicated sepsis (6.11 vs 1.97 and 1.95 ng/ml; *P *< 0.05, *P *< 0.02)	Endocan on ICU admission higher in nonsurvivors than patients still alive after 10 days (6.98 vs 2.54 ng/ml; *P *< 0.01), using a cutoff of 6.2 ng/ml, sensitivity and specificity are 75% and 84% respectively.
Bechard *et al*., [23]	2000	28	Patients with septic shock (8), healthy controls (20)	1992 ACCP/SCCM [1]	Higher in septic shock than healthy controls (7.815 vs 1.081 ng/ml; *P *= 0.0173)	

ACCP, American College of Chest Physicians; ALI, Acute Lung Injury; APACHE II, Acute Physiology and Chronic Health Evaluation II; ARDS, Acute Respiratory Distress Syndrome; ED, emergency department; MOF, Multiple Organ Failure; SAPS, Simplified Acute Physiology Score; SCCM, Society of Critical Care Medicine; SIRS, Systemic Inflammatory Response Syndrome; SOFA, Sequential Organ Failure Assessment

### Soluble ICAM-1

#### Association with sepsis

All studies comparing sICAM-1 in septic patients and healthy controls reported higher levels in septic patients [54,55,58,59,65,66,68]. sICAM-1 was also found to be significantly higher in sepsis than in patients with trauma [61,62,66,67], postoperative patients [55], patients with other forms of shock [52], and non-septic ICU patients [59,66,68]. One study reported that sICAM-1 levels were similar in septic patients and ICU patients without sepsis [65]. Two studies explicitly compared sICAM-1 in patients with sepsis and SIRS [53,68], but only one found higher sICAM-1 in sepsis [53]. Several studies observed that baseline sICAM-1 levels were similar in non-septic patients and healthy controls [55,59,66].

The association between sICAM-1 levels and sepsis severity was variable. Seven studies investigated this association, with four studies reporting higher sICAM-1 levels with increasing severity of sepsis [59,64,68,77] and three negative studies [53,61,65].

#### Association with clinical outcome

Eleven studies reported data on mortality. Five of these studies reported that increasing sICAM-1 levels correlated with mortality [55,58,59,68,77], but six studies found no such correlation [53,56,57,61,65,73]. One study found a trend towards increased mortality with increasing sICAM-1 levels over time [55].

Two studies evaluated the discriminative characteristics of sICAM-1 [51,58]. Weigand *et al*. reported that a sICAM-1 threshold of 800 ng/ml could differentiate survivors from non-survivors with a sensitivity and specificity of 74.1%, although this value was derived from a small sample of 14 post-surgical patients with relatively high mortality (50%) [58]. Shapiro reported on a group of 221 patients presenting to the emergency department with suspected infections, of which 208 had sepsis of varying severity. The presenting sICAM-1 value predicted mortality with an area under the receiver operating characteristic (ROC) curve of 0.72 (95% CI (0.57 to 0.870)). However, a cutoff value was not reported [77].

Several studies reported moderate to poor correlation of sICAM-1 with the degree of severity of illness or number of organ failures as defined by APACHE II, SOFA, Multiple Organ Failure Score and Simplified Acute Physiology Score [59,68,73,77].

One study reported varying kinetics of sICAM-1 according to age: In 30 patients with postoperative sepsis, Boldt *et al*. reported that older patients had higher sICAM-1 levels than younger patients (*P *< 0.05), and sICAM-1 tended to increase over time in older patients while decreasing over time in younger patients [60].

### Soluble VCAM-1 (sVCAM-1)

We identified 12 studies evaluating sVCAM-1 (see Table 3-Studies Evaluating sVCAM-1) in sepsis. These studies evaluated sVCAM-1 in emergency department patients [51], postoperative patients [55,63], patients admitted to ICU [56,62], critically-ill trauma patients [60] and patients with sepsis [64,65,69,78]. Three studies compared sVCAM-1 levels with healthy control groups [55,65,78].

#### Association with sepsis

Six studies reported that sVCAM-1 levels were significantly greater in patients with sepsis than in healthy controls [65,78], trauma patients [62,63], non-infected patients [77] and patients with multiple organ failure due to causes other than sepsis [64]. Four studies reported that sVCAM-1 levels effectively differentiated septic from non-septic patients [62-64,77], but one study reported sVCAM-1 levels were not significantly different between septic patients, postoperative patients and healthy controls [55]. One study reported higher sVCAM-1 levels in patients with shock due to sepsis compared to other forms of shock [52].

Three studies attempted to correlate sVCAM-1 with increasing sepsis severity [64,65,77]. Shapiro *et al*. found a moderate degree of correlation with severe sepsis with an area under the ROC curve of 0.60 (95% CI 0.52 to 0.69) [77]. Cowley *et al*. reported that baseline and peak values of sVCAM-1 were higher in ICU patients with severe sepsis than in uncomplicated sepsis [65]. Conversely, another study reported that sVCAM-1 was not different in septic patients with or without organ failure [64].

### Association with clinical outcome

Six of the 10 identified studies examined mortality outcomes, with 2 studies reporting an association between higher sVCAM-1 levels and mortality [55,77], and 4 studies showing no significant correlation with mortality in patients with ARDS [56], gram-positive sepsis [78], and septic patients admitted to ICU [65,69]. Hofer *et al*. found no correlation between baseline sVCAM-1 and mortality in septic patients but reported significantly higher sVCAM-1 levels at 48 and 120 hours in non-survivors compared to survivors.

Only one study addressed correlation of sVCAM-1 with clinical severity scores, and reported modest correlation with SOFA and APACHE II [77].

Two studies reported variability of sVCAM-1 in sepsis across different patient populations [64,69]. Presterl *et al*. investigated the difference of sVCAM-1 level in *Candida *sepsis compared to bacterial sepsis, and found that sVCAM-1 was higher in *Candida *sepsis at days 1, 7 and 14 [69]. Similar to sICAM-1, Endo *et al*. found higher sVCAM-1 levels with increasing age, and observed that the dynamics of serial sVCAM-1 were different in patients stratified by age. Specifically, sVCAM-1 values increased over the course of sepsis time in older patients and decreased in younger patients [64].

One study found that sVCAM-1 was not associated with left ventricular size or function in patients with sepsis or septic shock [76].

### Soluble E-selectin

Twenty-three studies were identified that evaluated sE-selectin as a biomarker in sepsis (see Table 4-Studies Evaluating sE-selectin-1).

#### Association with sepsis

The majority of identified studies reported higher levels of sE-selectin in sepsis compared to healthy controls or other patient groups without sepsis. Ten studies specifically reported significantly elevated sE-selectin levels in sepsis when compared with healthy controls [54,58,59,65,66,71,72,78-80]. Geppert *et al*. reported higher sE-selectin levels in patients with SIRS following cardiopulmonary resuscitation compared to controls [74]. sE-selectin was also reported to be significantly higher in septic patients compared to trauma patients [62,63,66], ICU controls [59,79], patients with infection but without systemic sepsis [61,77], patients with shock from other causes [52], and patients with multiple organ failure without infection [64]. Hynninen *et al*. concluded that sE-selectin values were not statistically different in patients with severe sepsis from those with severe acute pancreatitis [70].

#### Association with clinical outcome

The reported association of sE-selectin and disease severity has been inconsistent. Five studies showed a correlation between the marker and increasing sepsis severity [59,61,65,77,79], although three studies did not find a significant correlation [64,71,73].

Thirteen of the identified studies evaluated the association between sE-selectin and mortality, with nine studies reporting a significant positive correlation [59,61,69,73-75,77-79] and four studies reporting no correlation [56,58,65,70]. Among the studies reporting positive association, there was significant heterogeneity in the strength and type of association. One study of ICU patients with severe sepsis and septic shock reported that baseline sE-selectin-1 levels were higher in non-survivors than survivors, but the difference existed only for the first three days of sepsis [59]. In contrast, two other studies demonstrated a more persistent divergence of sE-selectin-1 between survivors and non-survivors of sepsis: Knapp *et al*. reported that sE-selectin-1 remained significantly elevated in non-survivors compared to survivors throughout the first seven days of sepsis [78], while Egerer reported that sE-selectin peaked in survivors of sepsis on the second day and decreased thereafter, whereas it continued to rise in patients who subsequently died [61]. One other study found that sE-selectin-1 predicted mortality in patients presenting to the emergency department with suspected infections, with an area under the ROC curve of 0.65 [51].

Only a few studies examined correlation between sE-selectin-1 and clinical severity of illness scores, and none found strong correlations. Shapiro *et al*. showed that sE-selectin correlated modestly with SOFA and APACHE-II [51]. Hynnien *et al*. reported that levels of sE-selectin were higher in patients with a SOFA score ≥ 10 compared to individuals with a score less than 10 [70]. sE-Selectin was also reported to correlate moderately or poorly with SAPSII [59,73] and MOF score [59,79].

Three studies evaluated variability in sE-selectin levels in different patient groups [60,69,79]. Boldt *et al*. showed sE-selectin levels in septic patients increased across age groups [60]. Cummings *et al*. showed higher levels in bacteremic sepsis than in non-bacteremic sepsis [79], and Presterl *et al*. found higher levels of sE-selectin in bacterial sepsis than in *Candida *sepsis [69].

### Endocan

Two prospective observational studies were identified evaluating endocan as a biomarker in sepsis [23,53] (see Table 5-Studies Evaluating Endocan).

#### Association with sepsis

Both studies reported that serum endocan was increased in septic patients. Schepereel *et al*. reported in their prospective study that endocan levels were higher in patients with sepsis than in patients with SIRS or healthy controls [53]. Bechard *et al*. showed that endocan levels were higher in patients with septic shock than in healthy controls [23].

#### Association with clinical outcome

Scherpereel *et al*. reported that mean endocan levels were higher in patients with septic shock than in patients with severe sepsis or sepsis. Furthermore, endocan levels measured at ICU admission were higher in non-survivors than in patients who were alive at 10 days. Using a threshold of 6.2 ng/ml, the sensitivity and specificity of endocan for predicting mortality were 75% and 84% respectively [53].

#### Mediators of permeability and vasomotor tone

We identified seven studies that examined soluble VEGF (see Table 6-Studies evaluating VEGF), two studies examining soluble FLT (Table 7-Studies Evaluating sFLT) and four studies examining endothelin-1 as biomarkers in sepsis (see Table 8-Studies Evaluating Endothelin-1). All but two were prospective studies, with two secondary analyses of previously conducted cohort studies [45,81]. Patients recruited were emergency room patients with suspected infection [51,77] or ICU patients [42,45,51,81-88].

**Table 6:** Studies evaluating VEGF

Study	Year	N	Population	Standard criteria for SIRS/sepsis	Association with sepsis	Other outcomes
Shapiro *et al*., [77]	2008	83	ED patients with septic shock (17), suspected infection without shock (66), and non-infected controls	Suspected infection based on treating clinician	VEGF levels higher in septic shock and infected patients without shock compared with non-infected controls (*P *< 0.01)	VEGF correlated with APACHE-II score at presentation (*P *= 0.01)
Karlsson *et al*., [82]	2008	280	Septic ICU patients (250) and healthy controls (30)	1992 ACCP/SCCM [1]	VEGF levels higher in severe sepsis compared with healthy controls at 0 and 72 h (*P *= 0.029, 0.003, respectively)	VEGF lower in non-survivors at 0 and 72 h (*P *= 0.012, 0.009, respectively), no correlation with SOFA scores
Kumpers *et al*., [42]	2008	72	Medical ICU (43) and healthy controls (29)	2003 ACCP/SCCM [2]	VEGF levels lower in non-septic and septic patients compared with healthy controls (*P *< 0.0001)	No association with severity of sepsis
Van der Heijden *et al*., [45]	2008	112	Mechanically ventilated patients with sepsis (24) and without (88)	1992 ACCP/SCCM [1]	VEGF levels higher in patients with sepsis than without sepsis (63.6 vs 20.7 pg/ml, *P *= 0.012)	VEGF trended higher in patients compared with controls (*P *= 0.268); No association with incidence of ALI/ARDS
Van der Flier *et al*., [83]	2005	58	Severe sepsis (18) and healthy controls (40)	1992 ACCP/SCCM [1]	VEGF levels elevated in sepsis compared with healthy controls (134 vs 55 pg/ml, *P *< 0.001)	VEGF correlated with mortality (*P *= 0.018)
Yang *et al*., [101]	2011	101	81 pneumonia and septic shock 20 pneumonia without organ dysfunction	1992 ACCP/SCCM [1]	VEGF levels lower in septic shock vs. pneumonia (*P *= 0.005)	Day 1 VEGF did not discriminate survivors from non-survivors (*P *= 0.46)
Rafat *et al*., [84]	2007	62	Medical ICU with sepsis (32), without sepsis (15), and healthy controls (15)	1992 ACCP/SCCM [1]	VEGF levels elevated in septic compared with non-septic patients (1,351 vs 477 pg/ml, *P *< 0.01)	VEGF not correlated with mortality (*P *< 0.48)

ACCP, American College of Chest Physicians; ALI, Acute Lung Injury; APACHE II, Acute Physiology and Chronic Health Evaluation II; ARDS, Acute Respiratory Distress Syndrome; ED, emergency department; MOF, Multiple Organ Failure; SAPS, Simplified Acute Physiology Score; SCCM, Society of Critical Care Medicine; SIRS, Systemic Inflammatory Response Syndrome; SOFA, Sequential Organ Failure Assessment

**Table 7:** Studies evaluating sFLT

Study	Year	N	Population	Standard criteria for SIRS/sepsis	Association with sepsis	Other outcomes
Schuetz *et al*., [52]	2011	161	Patients with hypotension: 69 sepsis, 35 cardiac, 12 hemorrhagic, 12 unknown	Clinical classification based on clinical and microbiological data	sFlt-1 higher in sepsis compared to non-sepsis (*P *< 0.05) SFlt-1 independently associated with sepsis after adjustment for age, sex, blood pressure and mortality (*P *= 0.03) with AUC 0.70 for discrimination of sepsis from non-sepsis	
Shapiro *et al*., [77]	2008	83	ED patients with septic shock (17), suspected infection without shock (66), and non-infected controls	Suspected infection based on treating clinician	sFLT levels elevated with worsening disease: non-infected, suspected infection without shock, septic shock (159, 386 and 551 ng/dL, respectively, *P *< 0.01)	sFLT correlated with APACHE-II, SOFA scores upon presentation and at 24 h (*P *< 0.05 for all)
Shapiro *et al*., [51]	2010	221	ED patients with sepsis without organ dysfunction (71), severe sepsis without shock (66), septic shock (71), and non-infected controls (13)	1992 ACCP/SCCM [1]	sFLT levels elevated in septic shock compared with non-infected controls (243 vs 41 ng/ml, *P *< 0.001)	sFLT correlated with SOFA, APACHE-II, lactate; Predicted severe sepsis and mortality (AUC of 0.82 (95% CI 0.76 to 0.88), 0.91 (95% CI 0.87 to 0.95))

ACCP, American College of Chest Physicians; ALI, Acute Lung Injury; APACHE II, Acute Physiology and Chronic Health Evaluation II; ARDS, Acute Respiratory Distress Syndrome; ED, emergency department; MOF, Multiple Organ Failure; SAPS, Simplified Acute Physiology Score; SCCM, Society of Critical Care Medicine; SIRS, Systemic Inflammatory Response Syndrome; SOFA, Sequential Organ Failure Assessment

**Table 8:** Studies evaluating Endothelin-1

Study	Year	N	Population	Standard criteria for SIRS/sepsis	Association with sepsis	Other Outcomes
Schuetz *et al*., [81]	2007	95	Consecutive ICU admissions with SIRS, sepsis, septic shock	1992 ACCP/SCCM [1]	Endothelin-1 rises with sepsis, septic shock, compared with SIRS (64.3, 131.6, 23.1 pmol/L, respectively; *P *< 0.01 between SIRS and sepsis, *P *< 0.05 between sepsis and septic shock)	Endothelin-1 not correlated with mortality (p = 0.87)
Piechota *et al*., [85]	2007	20	Medical ICU patients with sepsis	1992 ACCP/SCCM [1], severity graded by procalcitonin and C-reactive protein level	Endothelin-1 correlates with CRP and PCT levels as estimates of level of sepsis severity (*P *< 0.05 for both)	Endothelin-1 correlates with SOFA score (p < 0.001)
Weitzberg *et al*., [86]	1991	16	Sepsis (6) and healthy controls (10)	Bone *et al*., [102]	Endothelin-1 elevated in sepsis compared with healthy controls (11.3 vs. 2.4 pmol/l, *P *< 0.01)	n/a
Furian *et al*. [76]	2011	45	Patients admitted to ICU with severe sepsis or septic shock	1992 ACCP/SCCM [1]		Endothelin-1 levels associated with left ventricular and right ventricular function. (p = 0.002)
Pittet *et al*., [87]	1991	40	Sepsis (14), post-operative cardiac surgery (15) and healthy controls (11)	Bone *et al*., [102]	Endothelin-1 elevated in septic patients compared with healthy controls (19.9 vs 6.1 pg/ml, *P *< 0.0001)	Endothelin-1 inversely correlated with cardiac index (p < 0.005); correlated with APACHE-II scores (p < 0.01)

ACCP, American College of Chest Physicians; ALI, Acute Lung Injury; APACHE II, Acute Physiology and Chronic Health Evaluation II; ARDS, Acute Respiratory Distress Syndrome; ED, emergency department; MOF, Multiple Organ Failure; SAPS, Simplified Acute Physiology Score; SCCM, Society of Critical Care Medicine; SIRS, Systemic Inflammatory Response Syndrome; SOFA, Sequential Organ Failure Assessment

### Soluble VEGF

Four studies reported a positive association with sepsis, with higher levels in septic patients compared with non-septic critically ill patients [77,83,84] and healthy controls [82]. In contrast, Van der Heijden *et al*. did not find a significant difference in soluble VEGF between septic and non-septic ICU patients [45] and Kumpers *et al*. reported lower serum VEGF levels in patients with sepsis compared to healthy controls [42]. Van der Flier *et al*. reported significantly elevated VEGF levels in non-survivors compared with survivors [83], in contrast to Karlsson *et al*. who reported significantly lower VEGF levels in non-survivors [82].

### Soluble FLT (sFlt)

Both studies reporting sFLT were prospective studies from the same centre, studying emergency room patients with suspected infections, with non-infected patients serving as controls. There was some overlap between the two studies, with some patients reported in both cohorts. sFLT was shown to be elevated with increasing severity of illness [77], and was also predictive of severe sepsis and mortality, both upon presentation and longitudinally during hospitalization [51].

### Endothelin-1

Two studies reported that endothelin-1 was significantly elevated in patients with sepsis compared with healthy controls [86,87]. An additional two studies reported a correlation with severity of illness as defined by other biomarkers [85] or ACCP/SCCM criteria [81]. There was no documented association between endothelin-1 levels and mortality in the one study that examined this outcome [81].

### Mediators of coagulation

We identified 14 relevant studies studying von Willebrand Factor (vWF) and sepsis (see Table 9-Studies Evaluating von Willebrand Factor). All studies reported assays of either VWF:Ag and/or VWF:RCo activity. Four studies presented data on ADAMTS13 (see Table 10-Studies Evaluating ADAMTS13), which reported either ADAMTS13 antigen levels or ADAMTS13 activity.

**Table 9:** Studies evaluting von Willebrands factor

Study	Year	N	Population	Standard criteria for SIRS/sepsis	Association with sepsis	Other outcomes
Claus *et al*., [89]	2009	63	ICU patients with severe sepsis (11), non-elective cardiac surgery (22), elective cardiac surgery as ICU controls (24), and post-exercise as healthy controls (6)	1992 ACCP/SCCM [1]	VWF:Ag higher in patients with sepsis and post non-elective cardiac surgery than ICU controls (*P *< 0.05)	VWF:Ag shows tendency to discriminate survivors from nonsurivors
Bockmeyer *et al*., [90]	2008	57	ICU patients with sepsis (11), non-elective cardiac surgery (22), and elective cardiac surgery as ICU controls (24)	Not specified	VWF:Ag higher in sepsis and in non-elective cardiac surgery than ICU controls (both *P *< 0.001)	VWF:Ag correlated with mortality (*P *< 0.05)
van der Heijden *et al*., [45]	2008	112	Mechanically ventilated patients, with sepsis (24) and without (88)	1992 ACCP/SCCM [1]	VWF higher in patients with sepsis than without sepsis (*P *< 0.001)	VWF correlated with mortality (*P *= 0.006); VWF higher in those with ALI/ARDS than those without (*P *< 0.001)
Hovinga *et al*., [95]	2007	80	Medical and surgical ICU patients with severe sepsis or septic shock (40), and healthy controls (40)	1992 ACCP/SCCM [1]	VWF:Ag and VWF:RCO higher in sepsis than controls (*P *< 0.001)	VWF not correlated with disease severity, organ dysfunction, or mortality
Martin *et al*., [91]	2007	89	ICU patients with severe sepsis (30), sepsis-unrelated organ failure (29), and healthy controls (30)	1992 ACCP/SCCM [1]	VWF:Ag tends to differentiate severe sepsis from sepsis-unrelated organ failure (*P *> 0.05)	VWF:Ag not correlated with mortality
Scherpereel *et al*., [53]	2006	90	ICU patients with sepsis (63), SIRS (7), and healthy controls (20)	1992 ACCP/SCCM [1]	VWF higher in sepsis than SIRS (*P *< 0.02)	VWF correlated with mortality (*P *= 0.039)
Ware *et al*., [94]	2001	51	ICU patients with ALI, ARDS (45% due to sepsis)	Temperature > 38° or < 35°C, systolic blood pressure < 90 mmHg (or a drop of 20 mm Hg or more in the systolic blood pressure from baseline), both present for at least 2 h; AND a clinically identifiable source of infection [103]	VWF:Ag higher in patients with sepsis than those without (*P *< 0.05)	VWF correlated with mortality (*P *< 0.005); higher in those with longer duration of ventilation *P *< 0.005; not correlated with illness severity scores (SAPSII, Lung Injury Score)
Garcia-Fernandez *et al*., [92]	2000	80	ICU patients with SIRS and acute renal failure (40), and healthy controls (40)	1992 ACCP/SCCM [1]	VWF higher in SIRS than controls (*P *< 0.001)	
Bajaj *et al*., [97]	1999	60	Ward and ICU patients with ARDS (18), at risk of ARDS (15), and healthy controls (27)	Clinical diagnosis of sepsis	VWF does not differentiate patients with ARDS due to sepsis from other etiologies	VWF higher in ARDS (*P *< 0.001) and at risk ARDS (*P *< 0.01) compared to controls but did not differ significantly between these two groups
Kayal *et al*., [59]	1998	41	ICU patients with severe sepsis or septic shock (25), ICU controls (7), healthy controls (9)	1992 ACCP/SCCM [1]	VWF:Ag higher in sepsis than noninfected ICU controls and healthy controls (*P *< 0.0001); higher in septic shock than those without septic shock (*P *< 0.01)	VWF:Ag correlated with mortality (*P *< 0.01); correlated with SAPS and MOF score (r = 0.57, *P *< 0.01 for MOF)
Moss *et al*., [66]	1996	66	ICU patients with sepsis (19), trauma (36), healthy controls (11)	Clinical diagnosis of sepsis	VWF:Ag higher in septic patients than trauma patients and controls (both *P *< 0.001)	
Moss *et al*., [98]	1995	96	Hospitalized patients at risk of ARDS, including sepsis (30)	Clinical diagnosis of sepsis		VWF:Ag not predictive of the development of ARDS
Lorente *et al*., [93]	1993	48	ICU patients with septic shock	1992 ACCP/SCCM [1]		VWF:Ag not predictive of mortality
Rubin *et al*., [96]	1990	45	ICU patients with nonpulmonary sepsis	Clinical diagnosis of sepsis		VWF:Ag correlated with mortality (*P *< 0.005) and ALI (*P *< 0.01)

ACCP, American College of Chest Physicians; ALI, Acute Lung Injury; APACHE II, Acute Physiology and Chronic Health Evaluation II; ARDS, Acute Respiratory Distress Syndrome; ED, emergency department; MOF, Multiple Organ Failure; SAPS, Simplified Acute Physiology Score; SCCM, Society of Critical Care Medicine; SIRS, Systemic Inflammatory Response Syndrome; SOFA, Sequential Organ Failure Assessment

**Table 10:** Studies evaluating ADAMTS13

Study	Year	N	Population	Standard criteria for SIRS/sepsis	Association with sepsis	Other outcomes
Claus *et al*., [89]	2009	63	ICU patients with severe sepsis (11), non-elective cardiac surgery (22), elective cardiac surgery as ICU controls (24), and post-exercise as healthy controls (6)	1992 ACCP/SCCM [1]	ADAMTS13 activity lower in sepsis than ICU reference group (*P *< 0.001)	ADAMTS13 activity correlated with mortality (*P *< 0.05)
Bockmeyer *et al*., [90]	2008	57	ICU patients with sepsis (11), non-elective cardiac surgery (22), and elective cardiac surgery as ICU controls (24)	Not specified	ADAMTS13 activity lower in sepsis than ICU controls (*P *< 0.01)	ADAMTS13 activity correlated with mortality (*P *< 0.01)
Hovinga *et al*., [95]	2007	80	Medical and surgical ICU patients with severe sepsis or septic shock (40), and healthy controls (40)	1992 ACCP/SCCM [1]	ADAMTS13 activity lower in sepsis than healthy controls (*P *< 0.001)	ADAMTS13 activity not correlated with disease severity, organ dysfunction, or mortality
Martin *et al*., [91]	2007	89	ICU patients with severe sepsis (30), sepsis-unrelated organ failure (29), and healthy controls (30)	1992 ACCP/SCCM [1]	ADAMTS13 activity lower in severe sepsis than sepsis-unrelated organ failure (*P *< 0.05) and healthy controls (*P *< 0.05)	ADAMTS13 activity correlated with APACHE II (r = -0.66, *P *< 0.0001), number of organ failures (r = -0.66, *P *< 0.0001), and mortality (*P *= 0.02 by log rank test)

ACCP, American College of Chest Physicians; ALI, Acute Lung Injury; APACHE II, Acute Physiology and Chronic Health Evaluation II; ARDS, Acute Respiratory Distress Syndrome; ED, emergency department; MOF, Multiple Organ Failure; SAPS, Simplified Acute Physiology Score; SCCM, Society of Critical Care Medicine; SIRS, Systemic Inflammatory Response Syndrome; SOFA, Sequential Organ Failure Assessment

### Von Willebrand factor (vWF)

#### Association with sepsis

Eight studies examined the capability of circulating vWF levels to differentiate patients with sepsis from patients with other illnesses. Two studies found that vWF levels were significantly higher in septic patients compared to patients with systemic inflammation from other causes [89,90], other non-septic critically-ill patients [45,53,90], and healthy controls [59,89]. Two studies reported higher levels in patients with sepsis than in patients with SIRS or healthy controls, but the differences did not reach statistical significance [91-93]. In a cohort of patients with ALI/ARDS, Ware *et al*. reported that vWF was significantly increased in septic patients compared with those without sepsis (*P *< 0.05) [94].

Hovinga *et al*. in a secondary analysis of a clinical trial, reported that vWF activity was significantly higher in septic patients than in healthy controls, but vWF was not correlated with sepsis severity or survival [95]. Two other studies found a significant correlation between VWF and sepsis severity [59,94].

#### Association with clinical outcome

Four studies looked at its correlation with ALI/ARDS, with two studies showing its ability to differentiate those with ALI/ARDS from those without [45,96], and two studies showing that it is not predictive of ALI/ARDS [97,98].

Ten of the identified studies presented mortality data, with six studies showing a significant correlation of vWF with mortality [45,53,59,90,94,96], with one study reporting a plasma vWF:Ag of 450% the upper normal limit predicted death with a sensitivity of 44% and a specificity of 91% [94]. Four studies did not find a significant correlation with mortality [89,91,93,95].

### ADAMTS13

Three studies showed that ADAMTS13 is significantly lower in sepsis than other critically ill nonseptic patients [89-91]. One study showed significant correlation with disease severity [91], while a second did not [95]. Three studies showed ADAMTS13 levels correlated with mortality [89-91], although one study did not find a significant correlation [95].

## Discussion

We report a comprehensive and exhaustive systematic review of biomarkers reflecting endothelial activation for the diagnosis, triage and prognostication of sepsis in humans. The reviewed literature demonstrates positive associations between multiple EC-derived molecules and sepsis, supporting the critical role of EC activation in the septic response. Multiple other studies also reported positive associations for mortality and severity of illness, although these results were less consistent than for sepsis *per se*. Very few studies, however, reported thresholds or receiver operating characteristics that would establish these molecules as clinically-relevant biomarkers in sepsis.

Of the potential biomarkers reviewed, the angiopoeitin-1/2 system may hold the most promise. Multiple studies reported consistent associations between elevations in circulating Ang-2 levels and sepsis in varied samples of critically ill patients. All studies evaluating Ang-2 used standard sepsis definitions, with consistent association between Ang-2 levels and sepsis, as well as relatively consistent associations between Ang-2 and other clinical outcomes. The strength of association is also supported in the identified studies by: (1) a demonstrable dose-response relationship with higher Ang-2 levels in severe sepsis and organ dysfunction, and increasing with increasing severity of illness, and (2) a temporal progression with Ang-2 levels increasing over time in those patients who developed sepsis and in patients with increasing severity of sepsis as defined by SIRS, sepsis and septic shock. Unfortunately, no studies provided a cut point or threshold that would make Ang-2 clinically useful as a biomarker in the diagnosis or stratification of patients presenting with presumed sepsis.

One general limitation with all of the identified studies is the lack of standardized assays for the studied molecules. Very few studies reported threshold values for prognostic analysis or receiver operating characteristics of the potential biomarkers. Furthermore, almost all studies were either single centre or single laboratory, and most assays were non-standardized ELISAs, and thus the absolute values reported in each study may vary according to the type of assay, as well as the type of sample used (for example, plasma vs. serum). These issues led to important limitations in the generalizability and strength of inference that can be drawn from the identified studies. Where possible, we have reported absolute values in the tables to allow readers to appreciate the scope of variation, as well as absolute differences in levels between groups.

There are several limitations to our study. We searched for known endothelial-derived markers by name, and it is possible that other novel markers were missed. We attempted to address this limitation by hand-searching the reference list of identified studies to include all relevant studies of selected endothelial-derived markers. Many of the identified publications are single-centre studies or retrospective analyses of previously collected specimens, which limit generalizability to other jurisdictions and populations. As previously mentioned, lack of standardization in the reported assays makes quantitative comparison of a biomarker across studies impossible, and thus we can only report similarities in the direction and relative magnitude of association across studies.

The identified studies were most commonly small prospective or retrospective cohort studies evaluating levels of a potential biomarker in patients with sepsis and a comparative control group. Almost all studies used established consensus criteria for the definition of sepsis to limit misclassification of patients. There was significant heterogeneity in patient populations across studies, however, including patients with presumed sepsis identified in any one of the emergency department, medical ward and medical, surgical and trauma intensive care units. It is conceivable that the receiver operating characteristics of any given biomarker may vary according to the differential inflammatory state, concurrent injuries and pathophysiology of these different patient groups.

If EC-derived biomarkers are to become clinically useful, future work will require standardization of analytical techniques and rigorous evaluation of receiver operating characteristics to define the role and reliability of these molecules. Although some recent studies reported receiver operating characteristics or threshold biomarker levels, the lack of standard assays limits the interpretation and clinical utility of these efforts. Future work must include: (1) the description of the operating characteristics of biomarkers, (2) the use of explicitly defined threshold serum levels, (3) measured with a standardized assay.

It may be impossible to achieve the high degree of sensitivity and specificity required for clinical diagnosis with a single biomarker assay, and a multiplexed combination of markers may be necessary to improve predictive value and clinical utility of biomarkers. Careful selection and combinations of biomarkers with relative specificity to disease states (for example, the observed association between Ang-2 and ARDS/pulmonary leak, or the differential association of sVCAM and sE-Selectin in fungal sepsis) would be one way of improving the clinical utility of these novel molecules. Following identification of useful serum biomarker thresholds with standard assays, we speculate that evaluation of multiplexed biomarker panels may prove useful as a diagnostic strategy.

Given the epidemiologic rise of sepsis in both the developed [99] and developing world [100], novel diagnostics and therapeutics for sepsis are urgently needed, and endothelial-derived biomarkers will likely play a crucial role.

## Conclusions

We report a systematic review of the published literature and findings that multiple molecules reflecting endothelial activation are correlated with the presence of sepsis in humans. We also found variable degrees of correlation between biomarkers and other clinical outcomes. The clinical utility or application of these molecules as biomarkers in sepsis, however, is limited by a lack of standardization in analytical assays, a lack of data regarding receiver operating characteristics and, in the few cases where thresholds have been reported, a lack of validation in representative patient populations.

## Key messages

• Multiple molecules reflecting endothelial activation are correlated with the presence of sepsis in humans and other clinically important outcomes.

• The clinical utility or application of these molecules as biomarkers in sepsis; however, is limited by a lack of standardization in analytical assays, a lack of data regarding receiver operating characteristics and a lack of validation.

• The consistent association with sepsis, demonstrable dose-response relationship, and temporal progression in patients who develop sepsis make Angiopoietin-2 an attractive potential biomarker in sepsis.

• Future research should focus on standardization of assays and identification of cut points or thresholds that make biomarkers clinically useful in the diagnosis or stratification of patients presenting with presumed sepsis.

• Evaluation of multiplexed panels with biomarkers of differential response characteristics may prove useful as a diagnostic strategy.

## Abbreviations

ACCP: American College of Chest Physicians; ADAMTS13: a disintegrin and metalloproteinase with a thrombospondin type 1 motif, Member 13; ALI: Acute Lung Injury; Ang 1/2: Angiopoeitin 1/2; APACHE II: Acute Physiology and Chronic Health Evaluation II; ARDS: Acute Respiratory Distress Syndrome; EC: endothelial cell; ED: emergency department; ICU: intensive care unit; MOF: Multiple Organ Failure; SAPS: Simplified Acute Physiology Score; SCCM: Society of Critical Care Medicine; s-Flt: soluble Flt (soluble Vascular Endothelial Growth Factor receptor); sICAM-1: Intercellular Adhesion Molecule-1; SIRS: Systemic Inflammatory Response Syndrome; SOFA: Sequential Organ Failure Assessment; sVCAM-1: Vascular Cell Adhesion molecule-1; VEGF: Vascular Endothelial Growth Factor; vWF: von Willebrand Factor; vWFA: von Willebrand Factor antigen; vWFRCo: von Willebrand Factor ristocetin cofactor.

## Competing interests

The authors declare they have no competing interests.

## Authors' contributions

KX conceived of the study, participated in study design, participated in literature review and data extraction, and drafted the initial manuscript. WCL conceived of the study, participated in study design and provided critical revisions to the manuscript for intellectual content. JMS participated in study design, participated in literature review and data extraction, drafted the initial manuscript and provided critical revisions to the manuscript for intellectual content. SM participated in the literature review and data extraction, and drafted the initial manuscript. All authors participated in data synthesis and interpretation of results. All authors read and approved the final manuscript.

## Supplementary Material

Additional file 1 **Search Strategy**. Click here for file
