# Supplementary material for: Clinical utility of biomarkers of endothelial activation in sepsis-a systematic review
Source: Crit Care. 2012 Jan 16;16(1):R7. doi: 10.1186/cc11145 (PMC3396237; doi:10.1186/cc11145)
Supplement: Additional file 1 — Search Strategy. [file cc11145-S1.DOC]

# Search Strategy

1. Sepsis
2. angiopoietin OR tie-2 OR soluble tie-2 receptor OR adamts 13 OR endocan OR esm-1 OR endothelial cell specific molecule 1 OR endothelial specific molecule OR elam-1 OR endothelial leukocyte adhesion molecule 1 OR e-selectin OR vcam-1 OR vascular cell adhesion molecule-1 OR icam-1 OR intercellular adhesion molecule-1 OR vegf OR vascular endothelial growth factor a OR soluble VEGFR OR soluble vascular endothelial growth factor receptor OR soluble FLT-1 OR sFlt-1 OR vwf OR von willebrand factor OR endothelin-1
3. (#1) AND (#2)
